# Serum Acylcarnitines Associated with High Short-Term Mortality in Patients with Alcoholic Hepatitis

**DOI:** 10.3390/biom11020281

**Published:** 2021-02-14

**Authors:** Bei Gao, Josepmaria Argemi, Ramon Bataller, Bernd Schnabl

**Affiliations:** 1 School of Marine Sciences, Nanjing University of Information Science and Technology, Nanjing 210044, China; wintergb@hotmail.com; 2 Department of Medicine, University of California San Diego, La Jolla, CA 92093, USA; 3 Pittsburgh Liver Research Center, Department of Medicine, Division of Gastroenterology, Hepatology and Nutrition, University of Pittsburgh Medical Center, Pittsburgh, PA 15260, USA; j.argemi@pitt.edu (J.A.); bataller@pitt.edu (R.B.); 4 Department of Medicine, VA San Diego Healthcare System, San Diego, CA 92161, USA

**Keywords:** lipidomics, acylcarnitine, random forest, carnitine system

## Abstract

Alcohol-related liver disease is one of the most prevalent liver diseases in the United States. Early stages of alcohol-related liver disease are characterized by accumulation of triglycerides in hepatocytes. Alcoholic hepatitis is a severe form of alcohol-related liver disease associated with significant morbidity and mortality. We sought to identify patients who are at greatest risk of death using serum lipids. First, we performed lipidomics analysis on serum samples collected from 118 patients with alcoholic hepatitis to identify lipid markers that are associated with high risk of death. Next, we performed gene set enrichment analysis on liver transcriptomics data to identify dysregulated lipid metabolism in patients who received liver transplantation. Finally, we built a random forest model to predict 30-day mortality using serum lipids. A total of 277 lipids were annotated in the serum of patients with alcoholic hepatitis, among which 25 were significantly different between patients in the deceased and alive groups. Five chemical clusters were significantly altered between the two groups. In particular, acylcarnitine cluster was enriched in the deceased group. Several hepatic lipid metabolism pathways were dysregulated in patients with alcoholic hepatitis who received liver transplantation. The mRNA expression of genes involved in the fatty acid transport into mitochondria and β-oxidation were also dysregulated. When predicting 30-day mortality in alcoholic hepatitis patients using serum lipids, we found that the area under the curve achieved 0.95. Serum lipids such as acylcarnitines may serve as biomarkers to identify alcoholic hepatitis patients at the greatest risk of death.

## 1. Introduction

Alcohol-related liver disease is a global public health burden. Alcohol consumption is associated with increased hepatic accumulation of triglycerides. Fatty liver can develop through many pathways, including increased de novo lipogenesis and fatty acid uptake, reduced fatty acid oxidation, and impaired secretion of very low-density lipoprotein [1,2,3]. Alcohol-induced hepatic steatosis is characterized as the initial step of the disease spectrum, which can progress to steatohepatitis, fibrosis, and cirrhosis. Alcoholic hepatitis is an acute-on-chronic liver disease with prominent cholestasis, and it is associated with significant morbidity and mortality [4].

Circulating lipids are altered in patients with alcohol-associated cirrhosis [5,6]. However, the lipidome in patients with alcoholic hepatitis has not been well studied. Although fatty acids are endogenously generated by the host in general, intestinal microbes also metabolize dietary lipids and affect host lipid composition [7,8,9,10]. The contribution of microbial lipids to alcohol-related liver disease was studied by Chen et al., who showed chronic ethanol feeding reduced the bacterial biosynthesis of saturated long-chain fatty acids in mice [11]. Saturated long-chain fatty acid supplementation stabilized the gut barrier and ameliorated ethanol-induced liver injury in mice [11]. Nevertheless, the way in which microbial lipid metabolism affects the host lipid composition in alcoholic hepatitis patients is not well understood.

Severe alcoholic hepatitis is associated with high mortality and treatment options are limited. Characterization of lipid signature for alcoholic hepatitis patients at the greatest risk of death is helpful in terms of better understanding the disease and for the development of therapeutic strategies. In the present study, we compared the serum lipidomic profile from alcoholic hepatitis patients who were deceased at day 30 and who remained alive at day 30. Our aim was to identify lipid signatures for alcoholic hepatitis patients who are at the greatest risk of death.

## 2. Materials and Methods

### 2.1. Patients

Alcoholic hepatitis patient cohort for lipidomics and metagenomics has been described [12,13,14,15]. Inclusion and exclusion criteria of patients with alcoholic hepatitis are provided in the Supplementary Information, as reported in our previous publication [14]. Alcoholic hepatitis patients were enrolled in different medical centers in Europe and North America that were part of the InTeam Consortium (ClinicalTrials.gov identifier number: NCT02075918) between June 2014 and May 2018. The clinical picture was consistent with alcoholic hepatitis in all patients. Liver biopsies were performed only if it was indicated as the routine clinical care for diagnostic purpose. Liver histology was in line with the diagnosis of alcoholic hepatitis for patients who underwent liver biopsy. Single organ failure was defined as described previously [16]. The protocol was approved by the ethics committee of each center, and written informed consent was obtained from each patient. 

### 2.2. Profiling of Serum Lipids

Serum samples were collected from 118 alcoholic hepatitis patients. Serum lipids were analyzed as published previously [17]. Detailed methods on sample preparation and data acquisition are provided in Supporting Information. LC–MS raw data files were converted to ABF files using ABF converter (https://www.reifycs.com/AbfConverter/ accessed on 15 February 2020) and then processed by MS-DIAL version 2.94 [18] and MS-FLO [19], as described in our previous study [17]. For compound identification, retention time *m/z* libraries and MS/MS spectra databases were used as uploaded to MassBank of North America.

### 2.3. Shotgun Metagenomics Analysis

Fecal DNA was extracted from stool samples in 73 patients with alcoholic hepatitis using FastDNA Spin Kit for Soil (MP-Biomedicals), as described in our previous publication [13]. Detailed methods are provided in the Supplementary Information. Linear discriminant analysis effect size (LEfSe) was used for the biomarker discovery [20]. 

### 2.4. Liver Transcriptomics Analysis

Patient cohort and transcriptomics analysis of liver biopsies were described previously [21]. Gene set enrichment analysis (version 4.0.3) was performed on the transcriptomics data acquired from 11 patients with non-severe alcoholic hepatitis and 11 patients who received liver transplantation [22,23]. Kyoto Encyclopedia of Genes and Genomes (KEGG) subset of canonical pathways (version 7.1) was used as gene sets database. A total of 1000 permutations were performed. DESeq2 (1.26.0) was used for differential expression analysis [24].

### 2.5. Statistical Analysis

Statistical analysis was performed using R (version 3.5.1). Mann–Whitney–Wilcoxon test was used for the comparison of serum lipids between 2 groups. Adjusted *p*-values were calculated using Benjamini–Hochberg procedure to control the false discovery rate. Principal component analysis plots and heatmap was generated using MetaboAnalyst 4.0 [25]. Spearman correlation was conducted to correlate serum lipids with clinical parameters. To test the diagnostic value of serum lipids, we built a random forest model to predict the 30-day mortality in patients with alcoholic hepatitis using serum lipidome. Synthetic minority oversampling technique (SMOTE) was used to oversample the minor class to obtain balanced data. Extra-trees classifier was used to select 5 variables from all annotated serum lipids on the basis of the feature importance. Random forest model was built using H2O platform (https://www.h2o.ai accessed on 15 February 2020). The dataset was split into training and test datasets (80:20 stratified splits) and stratified fivefold cross-validation was performed on the training set to choose the tuning parameters for the model. Multivariate Cox regression model was used to detect associations of AC (10:0) with 30-day mortality, which was adjusted for the antibiotic and steroid treatments. Patients lost to follow-up were censored at the day they were last seen alive. Maximally selected rank statistic was used to determine the optimal cut-off value that represents the maximum difference of two alcoholic hepatitis groups regarding 30-day survival [26]. Kaplan–Meier curves along with log-rank test were used to compare 30-day survival between 2 groups. 

## 3. Results

### 3.1. Alcoholic Hepatitis Patient Cohort

A total of 118 patients were included in this study. Patient characteristics are summarized in Table S1. At day 30 following hospital admission, 99 patients remained alive, while 19 patients were deceased. Patients in the deceased group showed significantly higher levels of creatinine and bilirubin, higher Model for End-Stage Liver Disease (MELD) score, and lower levels of albumin (Table S1).

### 3.2. Dysregulation of Serum Lipidome

**Figure 1:**
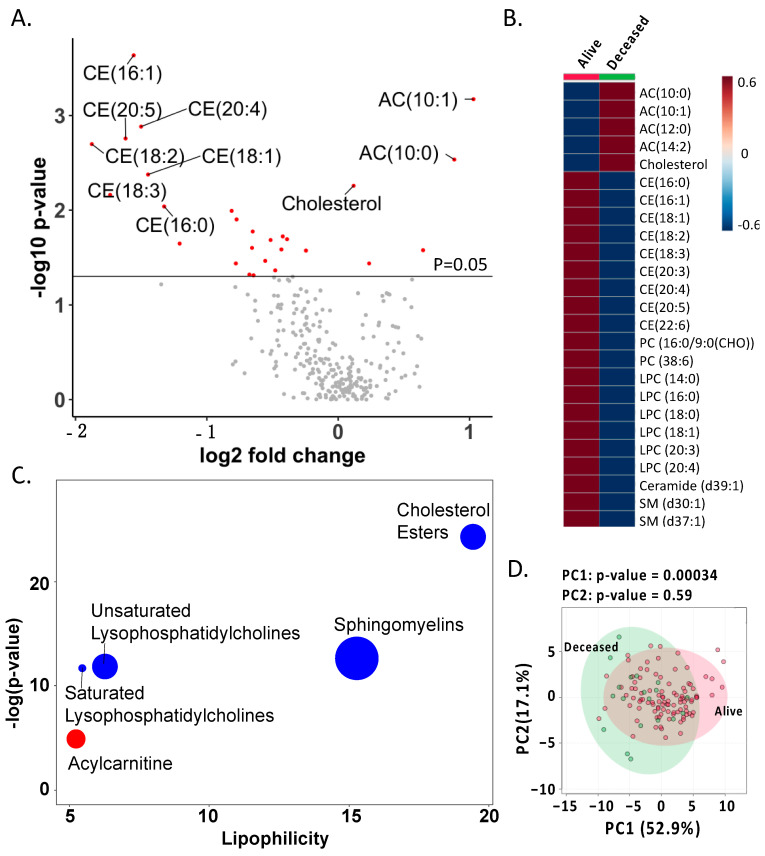
Serum lipidome in patients with alcoholic hepatitis. (**A**) Volcano plot of serum lipidome. Red dots: *p*-value < 0.05; gray dots: *p*-value > 0.05. Fold change: deceased group (*n* = 19)/alive group (*n* = 99) at day 30. (**B**) A total of 25 significant different serum lipids between the deceased group and alive group. (**C**) Chemical enrichment analysis of serum metabolome. Red: significantly increased lipid clusters in the deceased group; blue: significantly reduced lipid clusters in the deceased group. (**D**) Principal component analysis of serum metabolome in patients with alcoholic hepatitis.

A total of 277 lipids were annotated in the serum of patients with alcoholic hepatitis, among which 25 lipid markers were significantly different between patients who were alive at day 30 (alive group) and who were deceased at day 30 (deceased group, *p*-value < 0.05, Figure 1A,B). Five lipids were significantly increased in the deceased group, namely, acylcarnitine (AC 10:1), AC (10:0), AC (12:0), AC (14:2), and cholesterol. The remaining 20 lipids were significantly reduced in the deceased group. Chemical enrichment analysis showed that five chemical clusters were significantly altered, i.e., acylcarnitine was enriched in the deceased group and the other four clusters were reduced in the deceased group, namely, cholesterol esters (CE), sphingomyelins, and unsaturated and saturated lysophosphatidylcholine (LPC) (Figure 1C). Principal component analysis of the serum lipids showed that the alive group and deceased group were partially separated (PC1 *p*-value = 0.0003, Figure 1D). 

### 3.3. Association of Serum Lipids with Clinical Parameters

**Figure 2:**
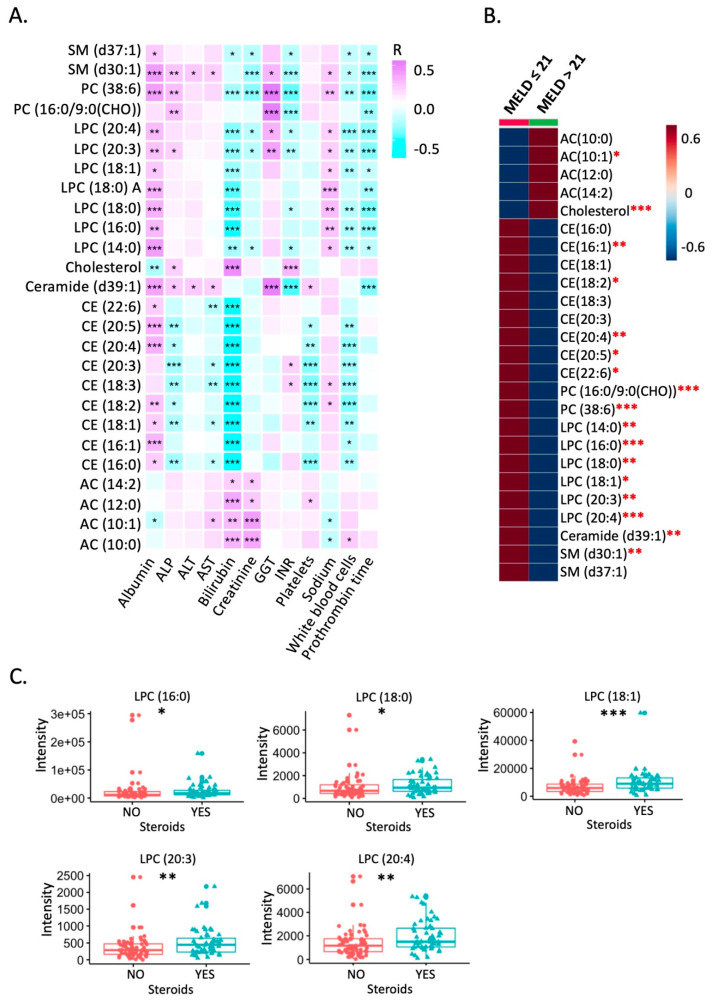
Correlation between serum lipid markers and clinical parameters. (**A**) Spearman correlation between 25 serum lipid markers and clinical parameters. (**B**) Different lipids in patients with MELD score ≤ 21 or >21. (**C**) The impact of steroid treatment on serum lipids. * *p*-value < 0.05; ** *p*-value < 0.01; *** *p*-value < 0.001.

To reveal the association between serum lipids and clinical parameters, we performed Spearman correlation analysis between the differentially expressed 25 serum lipid markers (Figure 1B) and 12 clinical parameters. The correlation results are shown in Figure 2A. Further, we assessed the statistical difference between patients with MELD score ≤ 21 and MELD score > 21. AC (10:1) and cholesterol were higher in patients with MELD score > 21, while 15 lipids were reduced in patients with MELD score > 21, including LPCs (Figure 2B). The serum level of five LPCs were higher in patients with steroid treatment (Figure 2C).

**Figure 3:**
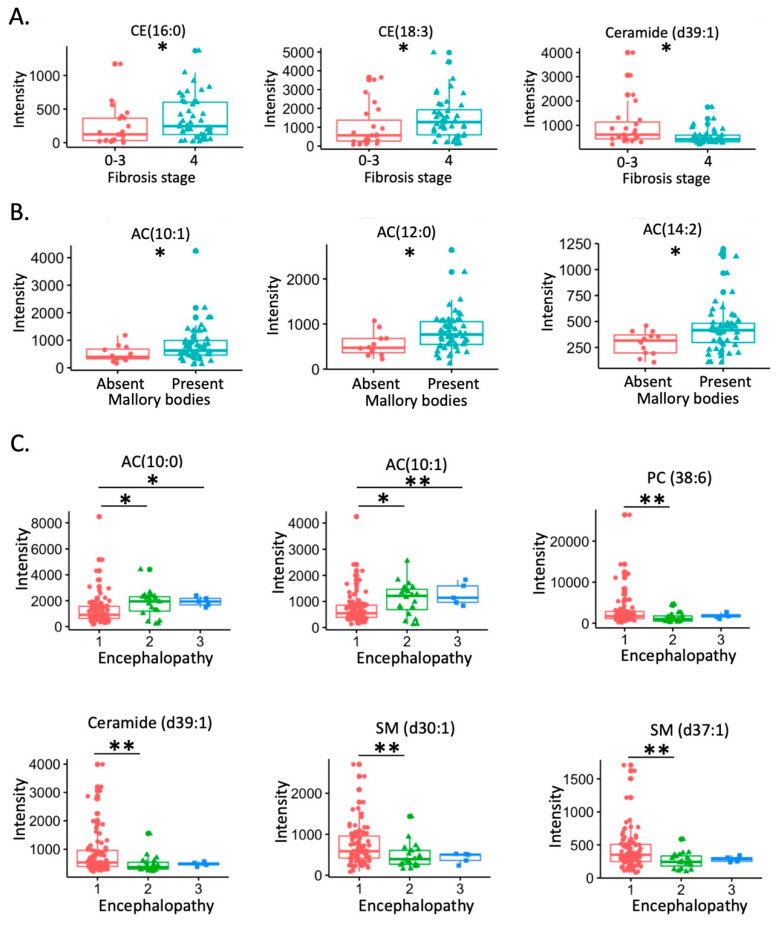
Changes of serum lipids. (**A**) Change of lipids with fibrosis stage. 0: no fibrosis; 1: portal fibrosis; 2: expansive periportal fibrosis; 3: bridging fibrosis; 4: cirrhosis. (**B**) Change of lipids with Mallory bodies. (**C**) Change of lipids with the status of encephalopathy. Encephalopathy: 1 no; 2 mild; 3 severe. *: *p* < 0.05; **: *p* < 0.01.

CE (16:0) and CE (18:3) were higher in alcoholic hepatitis patients with cirrhosis, while ceramide (d39:1) was lower in patients with cirrhosis (Figure 3A). AC (10:1), AC (12:0), and AC (14:2) were increased in patients with Mallory bodies compared with patients without Mallory bodies on liver biopsy (Figure 3B). AC (10:0) and AC (10:1) were increased in the patients with mild and severe encephalopathy compared with patients with no encephalopathy; meanwhile, phosphatidylcholine (PC 38:6), ceramide (d39:1), sphingomyelin (SM d30:1), and SM (d37:1) were reduced in patients with mild encephalopathy compared with patients without encephalopathy (Figure 3C). 

### 3.4. Microbial Mevalonate Pathway was Enriched in Patients with More Severe Alcoholic Hepatitis

**Figure 4:**
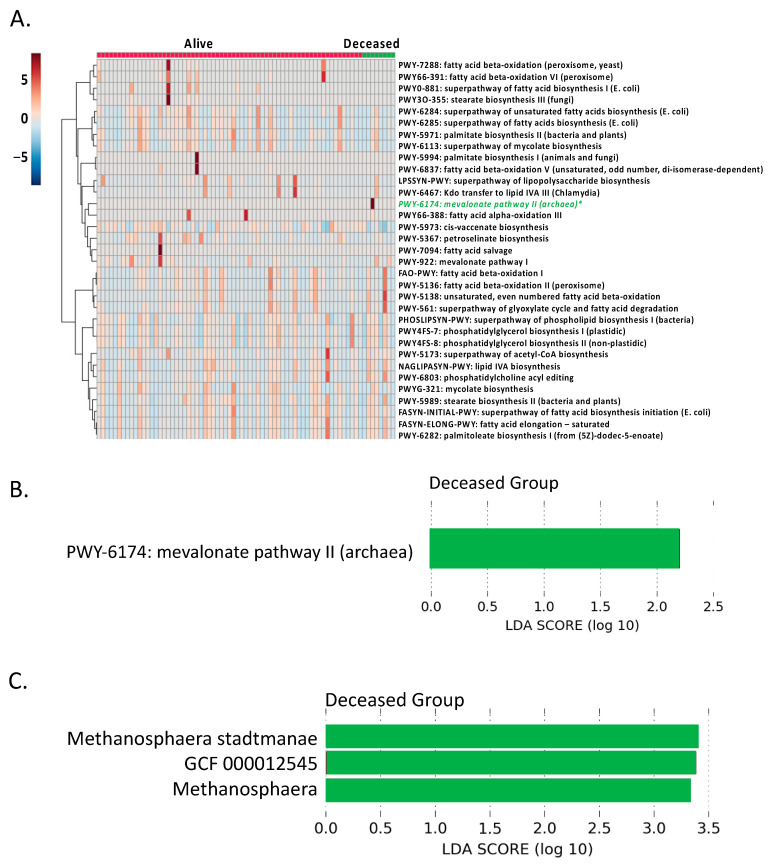
Microbial lipid metabolism. (**A**) Detected microbial lipid pathways in patients with alcoholic hepatitis. * Significant increased pathway in patients who were deceased at day 30 compared with patients who were alive at day 30. (**B**) PWY-6174: mevalonate pathway II (archaea) was enriched in patients who were deceased at day 30. (**C**) Enriched archaea in patients who were deceased at day 30.

To investigate the contribution of microbiota to host lipid metabolism, we identified 34 microbial pathways involved in lipid metabolism, including fatty acid biosynthesis and degradation, lipopolysaccharide biosynthesis, phospholipid biosynthesis, sphingolipid biosynthesis, and terpenoid biosynthesis (Figure 4A). LEfSe analysis showed one microbial pathway was significantly different between two patient groups, namely, mevalonate pathway II (archaea), which was enriched in patients who were deceased at day 30 (Figure 4B). Two archaea genera were detected in our patient cohort, namely, Methanobrevibacter and Methanosphaera. Methanosphaera was enriched in patients who were deceased at day 30, as well as Methanosphaera_stadtmanae, and a strain within Methanosphaera_stadtmanae, GCF 000012545 (Figure 4C). 

### 3.5. Transcriptomic Analysis Demonstrated Dysregulated Mitochondrial Lipid Metabolism in Liver of Patients with More Severe Alcoholic Hepatitis

**Figure 5:**
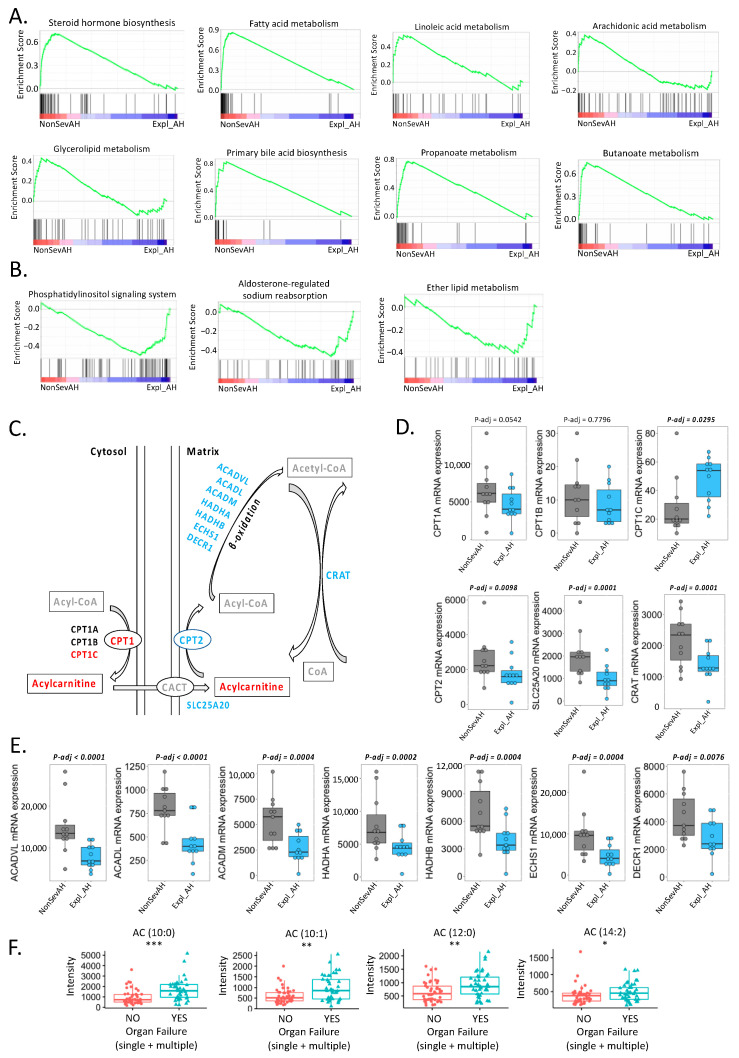
Gene set enrichment analysis of liver transcriptomics data from alcoholic hepatitis patients. (**A**) Enriched pathways in non-severe alcoholic hepatitis patients. (**B**) Enriched pathways in patients who received liver transplantation. (**C**) Carnitine transport system. Red: increased in patients who received liver transplantation; blue: reduced in patients who received liver transplantation; black: no significant change; gray: not detected. (**D**) Hepatic mRNA expression of genes involved in carnitine transport system in alcoholic hepatitis patients. (**E**) Hepatic mRNA expression of genes involved in fatty acid β-oxidation in alcoholic hepatitis patients. NonSevAH: non-severe alcoholic hepatitis patients. Expl_AH: alcoholic hepatitis patients who received liver transplantation. (**F**) Acylcarnitines were increased in patients who had organ failure. NonSevAH: non-severe alcoholic hepatitis patients. Expl_AH: alcoholic hepatitis patients who received liver transplantation. *: *p* < 0.05; **: *p* < 0.01; ***: *p* < 0.001.

To determine the contribution of hepatocytes to lipid metabolism, we performed gene set enrichment analysis on transcriptomic data acquired from liver biopsies and explants from a second patient cohort of alcoholic hepatitis [21]. We compared patients with non-severe alcoholic hepatitis (as defined by MELD < 21) with patients who received liver transplantation for severe alcoholic hepatitis. Eight pathways related to lipid metabolism were enriched in non-severe alcoholic hepatitis patients, including steroid hormone biosynthesis, fatty acid metabolism, linoleic acid metabolism, arachidonic acid metabolism, glycerolipid metabolism, primary bile acid biosynthesis, propanoate metabolism, and butanoate metabolism (Figure 5A). Three pathways associated with lipid metabolism were enriched in patients who received liver transplantation, namely, phosphatidylinositol signaling system, aldosterone-regulated sodium reabsorption, and ether lipid metabolism (Figure 5B). 

Since we found acylcarnitines to be higher in patients with more severe disease, we further assessed this metabolic pathway by targeted transcriptomic analysis. Transport of fatty acids across mitochondrial membrane relies on the carnitine system, components of which are dysregulated in the alcoholic hepatitis patients who received liver transplantation compared with the non-severe alcoholic hepatitis patients, including the upregulation of the carnitine palmitoyltransferase 1 (CPT1) isoform C and downregulation of CPT2 and the carnitine acetyltransferase (CRAT), which closes the carnitine cycle (Figure 5C,D). Meanwhile, downregulation of genes involved in fatty acid β-oxidation was also found in alcoholic hepatitis patients who received liver transplantation (Figure 5C,E). These results suggested that the disruption of carnitine system and fatty acid β-oxidation function in the mitochondria was associated with higher risk of death, in line with the accumulation of acylcarnitine in the serum (Figure 1B). In this cohort, 41 alcoholic hepatitis patients had organ failure, including 24 patients with single organ failure and 17 patients with multiorgan failure (Table S1). Increased serum level of acylcarnitines was found in these 41 patients who had organ failure (Figure 5F). 

### 3.6. Serum Lipidomic Signature Predicts 30-Day Mortality 

**Figure 6:**
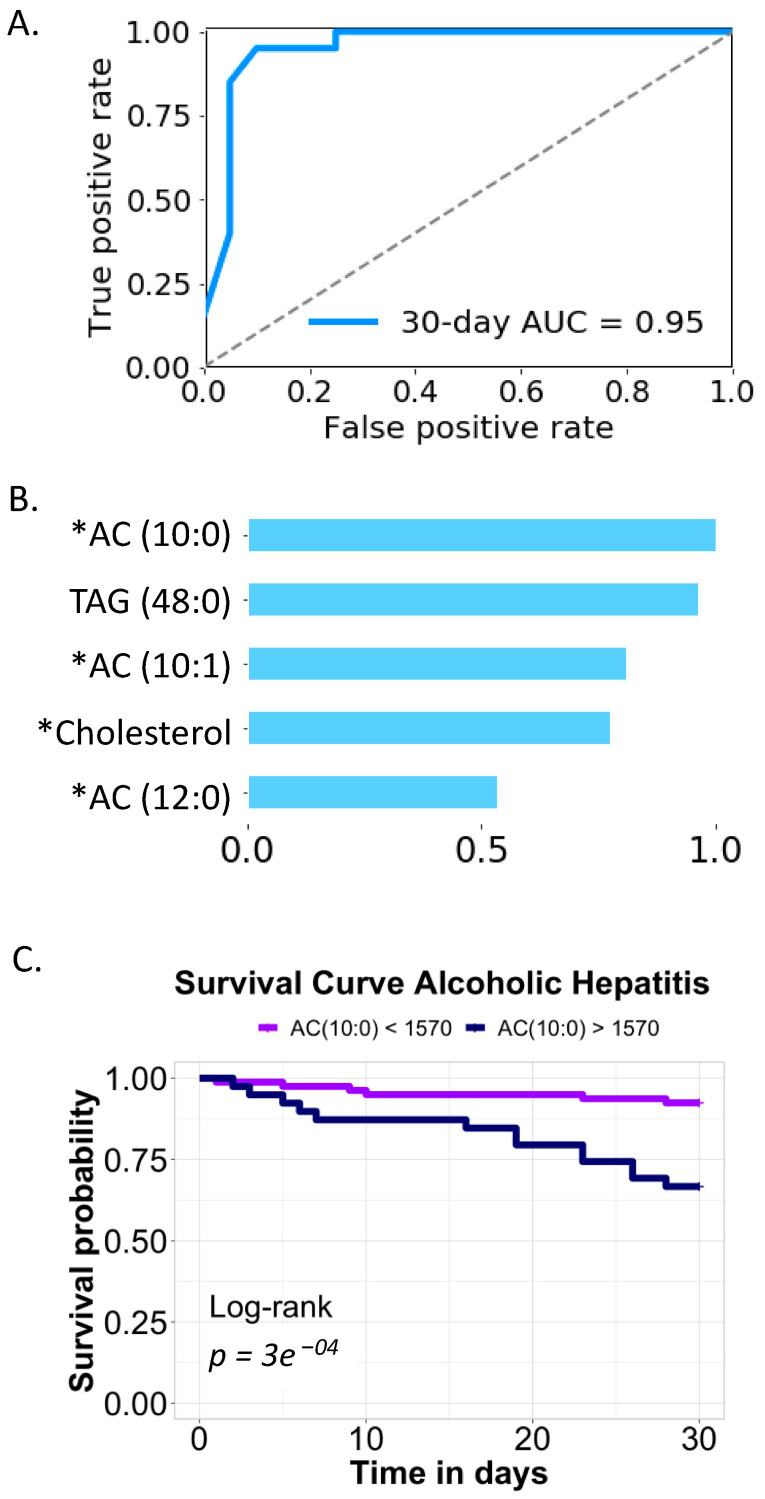
Thirty-day mortality prediction in patients with alcoholic hepatitis. (**A**) Random forest model. (**B**) Variable importance. * *p* < 0.05 between deceased and alive groups. (**C**) Survival curve based on the intensity of AC (10:0).

To test the predictive value of serum lipids, we built a random forest model to predict 30-day mortality in alcoholic hepatitis patients. During the feature selection process, the five most predictive lipids were selected using extra-trees classifier, including acylcarnitines. The area under the curve achieved 0.95 when predicting 30-day mortality for patients with alcoholic hepatitis (Figure 6A). The variable importance of the five selected serum lipids is shown in Figure 6B. Four of these lipids were significantly increased in patients of the deceased group, namely, AC (10:0), AC (10:1), cholesterol, and AC (12:0). Interestingly, although triacylglycerol (TAG) (48:0) was not significantly different between the two groups, it was valuable for prediction of 30-day mortality in patients with alcoholic hepatitis. Further, we evaluated the patients’ survival rate using the most predictive feature AC (10:0). Using maximally selected rank method, we found patients with serum level of AC (10:0) greater than an intensity of 1570 had a significantly lower 30-day survival compared with those with serum level lower than or equal to 1570 (Figure 6C).

## 4. Discussion

Although one of the features of alcohol-associated liver disease is hepatic steatosis, lipidomics has not been applied to study alcoholic hepatitis. In the present study, chemical enrichment analysis showed that five lipid clusters were significantly different between patients in the deceased group and in the alive group. In particular, acylcarnitine cluster was significantly enriched in deceased patients (Figure 1B). Acylcarnitines are esters of carnitine and fatty acids, serving as carriers to transport activated long-chain fatty acids into mitochondria. After mitochondrial translocation, acylcarnitines are converted to acyl-CoA, which enters the fatty acid β-oxidation pathway. In the present study, upregulation of CPT1C, downregulation of CPT2, and genes involving in fatty acid β-oxidation were found in patients who received liver transplantation, suggesting that mitochondrial function regarding the fatty acid transport and β-oxidation are damaged in the hepatocytes of these patients (Figure 5C). This is supported by our finding that serum acylcarnitines are higher in patients with Mallory bodies present on liver biopsy, which is a marker of hepatocyte damage (Figure 3B). Abnormal carnitine system and fatty acid β-oxidation function might lead to the accumulation of serum acylcarnitines in patients who had higher risk of death. The malfunction of mitochondria in the hepatocytes and accumulation of acylcarnitines in the systemic circulation might further contribute to the failure of other organs. As shown in Figure 5F, accumulation of serum acylcarnitines was found in patients who had organ failure. Abnormal carnitine/acylcarnitine profile was also reported in patients with acute liver failure [27]. In addition, blood level of long-chain acetylcarnitines were positively correlated with liver cirrhosis in patients [28,29]. 

Cholesteryl esters are the predominant form of cholesterol transport and storage. In this study, the serum level of cholesterol was increased in patients of the deceased group; meanwhile, cholesterol esters were reduced in patients of the deceased group (Figure 1B). This result suggests that cholesterol was inefficiently converted to cholesteryl ester in the blood of alcoholic hepatitis patients in the deceased group. The inefficient conversion of cholesterol to cholesterol esters was found in patients with cardiovascular disease [30]. In addition, gene set enrichment analysis showed that steroid hormone biosynthesis was enriched in non-severe alcoholic hepatitis patients (Figure 5A). Steroid hormones are derived from cholesterol [31]. Increased biosynthesis of steroid hormone might also contribute to the reduction of cholesterol in non-severe alcoholic hepatitis patients.

The way in which gut microbiota contributes to host lipid metabolism is under active investigation. It has been reported that sphingolipids produced by the gut bacteria enter host metabolic pathways and affect host lipid metabolism [10]. Gut microbiota also has the ability to produce metabolites derived from dietary polyunsaturated fatty acids [9]. Higher level of hydroxy fatty acids were found in pathogen-free mice compared with germ-free mice [8]. In the present study, mevalonate pathway II (archaea) was enriched in patients who were deceased at day 30 (Figure 4B). Archaea membrane lipids contain isoprenoid side chains, which are synthesized via mevalonate pathway [32,33]. Further studies are needed to evaluate if membrane lipids from archaea could pass the dysfunctional intestinal barrier, enter the portal vein, and affect the host lipid metabolism, such as hepatic cholesterol biosynthesis, in alcoholic hepatitis patients. In addition, gene set enrichment analysis showed that propanoate and butanoate metabolism in the liver was enriched in patients with non-severe alcoholic hepatitis (Figure 5A). Propanoate and butanoate are short-chain fatty acids mainly produced by the gut microbiota in the intestine [34]. The changes of propanoate and butanoate metabolism in the liver suggest an impact of the gut microbiota on host lipid metabolism.

In summary, we performed lipidomics analysis on serum of patients with alcoholic hepatitis and identified 25 serum lipid markers that were significantly different between patients in the deceased and alive groups. In particular, acylcarnitines were significantly increased in the deceased patients. Serum acylcarnitines are helpful for the prediction of short-term mortality. Our study provides valuable information for the identification of new drug targets and development of lipid-based therapeutic strategies for patients with alcoholic hepatitis.

## Supplementary Materials

The following are available online at https://www.mdpi.com/2218-273X/11/2/281/s1: Table S1: Characteristics of patients with alcoholic hepatitis. Supplementary Methods.
