# Supplementary material for: Serum Acylcarnitines Associated with High Short-Term Mortality in Patients with Alcoholic Hepatitis
Source: Biomolecules. 2021 Feb 14;11(2):281. doi: 10.3390/biom11020281 (PMC7917657; doi:10.3390/biom11020281)
Supplement: Supplementary file 1 [file biomolecules-11-00281-s001.pdf]

Supplementary Materials

Serum acylcarnitines associated with high short-term mortality in patients with alcoholic hepatitis

Table S1. Characteristics of patients with alcoholic hepatitis (n=118).

Clinical parameters	Alive Group (n=99)	Deceased Group (n=19)	*p*-value
Sex (male), n (%), n=117	61 (61.6%)	14 (73.7%)	0.4902
Age (years), n=116	49 (26-70)	51 (32-66)	0.1879
BMI (kg/m^2), n=98	26 (16-45)	27 (21-42)	0.3087
**Laboratory parameter**			
Creatinine (mg/dL), n=114	0.7 (0.3-5.5)	1.6 (0.6-5.5)	**0.0001**
Bilirubin (mg/dL), n=114	13.4 (2.5-51.8)	21.0 (3.5-39.5)	**0.0208**
AST (IU/L), n=114	122.0 (34.0-486.0)	144.5 (72.0-1858.0)	0.3294
ALT (IU/L), n=114	44.0 (15.0-174.0)	46.0 (25.0-404.0)	0.9288
Albumin (g/dL), n=112	2.5 (1.3-4.2)	2.1 (1.5-3.3)	**0.0092**
INR, n=114	1.8 (0.8-3.7)	1.9 (1.2-7.6)	0.1697
GGT (IU/L), n=62	249.5 (33.0-3145.0)	105.5 (33.0-1669.0)	0.3348
Platelet count (10^9/L), n=113	120.0 (13.0-447.0)	111.5 (12.2-355.0)	0.5149
Alkaline phosphatase (U/l), n=113	170.0 (21.2-511.0)	161.0 (85.0-478.0)	0.5855
Prothrombin time, s, n=99	23.2 (9.0-60.0)	24.1 (13.2-141.0)	0.7144
Sodium (mEq/L), n=114	134.0 (118.0-143.0)	132.0 (106.0-148.0)	0.3323
Encephalopathy, n=115			**0.0005**
1, n (%)	81 (84.4%)	9 (47.4%)	
2, n (%)	13 (13.5%)	7 (36.8%)	
3, n (%)	2 (2.1%)	3 (15.8%)	
Infections, n (%), n=94	18 (21.4%)	4 (4.0%)	0.3596
Organ failure, n (%), n=87	25 (35.7%)	16 (94.1%)	**< 0.0001**
Single Organ failure, n	17	7	
Liver, n	14	4	
Renal, n	3	1	
Respiratory, n	0	1	
Pancreas, n	0	1	
Multiorgan failure, n	8	9	
**Treatment at admission**			
Steroids, n (%), n=114	46 (47.9%)	8 (44.4%)	0.9892
Antibiotics, n (%), n=114	44 (45.8%)	12 (66.7%)	0.1721
Prophylactic antibiotics, n=114	24 (25%)	7 (36.8%)	0.3541
Proton pump inhibitors, n (%), n=51	4 (9.5%)	1 (11.1%)	**< 0.0001**
**Clinical scores**			
MELD, median (range), n=114	23.2 (7.6-43.0)	29.2 (21.2-57.5)	**0.0002**
MELD>21, n (%), n=88	70 (80%)	18 (20%)	**0.0273**
**Histology**			
Liver biopsy available, n (%), n=68	59 (87%)	9 (13%)	0.4041
Stage of fibrosis, n=67			0.4237
0, n (%)	1 (1.7%)	0 (0%)	
1, n (%)	2 (3.4%)	0 (0%)	
2, n (%)	6 (10.3%)	3 (33.3%)	

3, n (%)	10 (17.2%)	1 (11.1%)	
4, n (%)	39 (67.2%)	5 (55.6%)	
Mallory bodies, n (%), n=66			0.9645
0, n (%)	10 (17.2%)	2 (25%)	
1, n (%)	48 (82.8%)	6 (75%)	

Note: Values presented are median with range in parentheses for continuous variables, or number and percentage in parentheses for categorical variables. Wilcoxon rank sum test was used for the comparisons for continuous variables, and Chi-squared test was used for categorical variables. Percentages were calculated based on the actual number of patients in each group, when data were available. The number of subjects for which data were available is indicated in the first column. MELD, model for end-stage liver disease; PMN, polymorphonuclear infiltration. BMI, body mass index; AST, aspartate aminotransferase; ALT, alanine aminotransferase; INR, international normalized ratio; GGT, gamma-glutamyl transferase; Fibrosis stage, 0 no fibrosis, 1 portal fibrosis, 2 expansive periportal fibrosis, 3 bridging fibrosis, 4 cirrhosis; Mallory bodies, 0 absent, 1 present.

Methods

Inclusion and exclusion criteria of patients with alcoholic hepatitis

Inclusion criteria for alcoholic hepatitis were: 1 active alcohol use (> 50 g/day for men and > 40 g/day for women) in the last 3 months; 2 aspartate aminotransferase (AST) > alanine aminotransferase (ALT) and total bilirubin > 3 mg/dl in the past 3 months; 3 liver biopsy and/or clinical picture consistent with alcoholic hepatitis. Exclusion criteria were: 1 autoimmune liver disease (ANA > 1/320); 2 chronic viral hepatitis; 3 hepatocellular carcinoma; 4 complete portal vein thrombosis; 5 extrahepatic terminal disease; 6 pregnancy; 7 lack of signed informed consent.

Profiling of serum lipids

20 μL serum samples were extracted with 225 μL of −20 °C cold, degassed methanol and 750 μL of methyl tertiary butyl ether (MTBE, Sigma Aldrich, St. Louis, MO, USA). MTBE contained cholesteryl ester 22:1 as an internal standard. 16 internal standards were contained in methanol for quality control and retention time correction: ceramide (d18:1/17:0), cholesterol-d7, DG (12:0/12:0/0:0), DG (18:1/2:0/0:0), MG (17:0/0:0/0:0), LPC (17:0), LPE (17:1), palmitic acid-d3, PC (17:0/0:0), PC (12:0/13:0), PE (17:0/17:0), PE (17:1/0:0), PG (17:0/17:0), SM (d18:0/17:0), sphingosine C17, TG (17:0/17:1/17:0)-d5. 188 μL of liquid chromatography-mass spectrometry (LC-MS) grade water was added to induce phase separation, followed by centrifugation at 14,000 g for 2 min. 350 μL upper non-polar phase was collected, evaporated to dryness and then re-suspended in 9:1 methanol:toluene (v/v) which contained 50 ng/mL 12-[[(cyclohexylamino)carbonyl]amino]-dodecanoic acid (CUDA, Cayman Chemical, Ann Arbor, MI, USA) as an internal standard. Re-suspended samples were injected onto an Acquity UPLC CSH C18 (100 mm × 2.1 mm × 1.7 μm) column coupled to an Acquity VanGuard CSH C18 pre-column (5 mm × 2.1 mm × 1.7 μm, Waters, Milford, MA, USA) at 1.7 μL and 5 μL for ESI positive and negative modes, respectively. The column was maintained at 65 °C throughout the run. Mobile phase A consisted of 60:40 acetonitrile:water

(v/v) and mobile phase B consisted of 90:10 isopropanol:acetonitrile (v/v). Ammonium formate (10 mM) and 0.1% formic acid (Sigma–Aldrich, St. Louis, MO, USA) were used as modifiers for the positive mode and ammonium acetate (10 mM) (Sigma–Aldrich, St. Louis, MO, USA) was used as modifier for the negative mode.

The gradient was 0 min 15% B; 0-2 min 30% B; 2-2.5 min 48% B; 2.5-11 min 82% B; 11-11.5 min 99% B; 11.5-12 min 99% B; 12-12.1 min 15% B; and 12.1-15 min 15% B. The flow rate was 0.6 mL/min. Agilent 6530 QTOF was used for data acquisition with mass range 120-1700 m/z; capillary voltage ±3500 V; nozzle voltage ±1000 V; gas temperature 325 ℃; drying gas (nitrogen) 8 L/min; nebulizer gas (nitrogen) 35 psi; sheath gas temperature 350 ℃; sheath gas flow (nitrogen) 11 L/min and acquisition rate 2 spectra/s. MS/MS spectra were collected at a collision energy of 20 eV with an acquisition rate MS1 of 2 spectra/s and MS/MS of 4 spectra/s with 4 precursor ions per cycle. Mass accuracy was maintained by constant reference ion infusion (purine and HP-0921 in acetonitrile:water solution). Human plasma (BioIVT, Westbury, NY, USA) and method blank samples were injected at the beginning of the run and every ten samples throughout the run as quality control.

Shotgun metagenomics analysis

Whole-genome shotgun metagenomic sequencing was performed on Illumina HiSeq 4000 which generated 150bp paired-end reads. Quality control of raw sequencing reads was performed using KneadData version 0.7.2, followed by Metagenomic Phylogenetic Analysis 2 (MetaPhlAn2) version 2.7.7 [1] for the profiling of microbial species and HMP Unified Metabolic Analysis Network 2 (HUMAnN2) version 0.11.1 [2] for the profiling of microbial pathways in MetaCyc database.

References

1. Truong DT, Franzosa EA, Tickle TL, et al. MetaPhlAn2 for enhanced metagenomic taxonomic profiling. Nat Methods 2015;12:902-903.

2. Franzosa EA, McIver LJ, Rahnavard G, et al. Species-level functional profiling of metagenomes and metatranscriptomes. Nat Methods 2018;15:962-968.
